# The sensitivity and specificity of chest CT in the diagnosis of COVID-19

**DOI:** 10.1007/s00330-020-07347-x

**Published:** 2020-10-13

**Authors:** Anita Kovács, Péter Palásti, Dániel Veréb, Bence Bozsik, András Palkó, Zsigmond Tamás Kincses

**Affiliations:** Department of Radiology, Albert Szent-Györgyi Clinical Center, University of Szeged, Semmelweis u. 6, Szeged, 6725 Hungary

**Keywords:** COVID-19, Multidetector computed tomography, Real-time polymerase chain reaction, Sensitivity, Specificity

## Abstract

**Purpose:**

The identification of patients infected by SARS-CoV-2 is highly important to control the disease; however, the clinical presentation is often unspecific and a large portion of the patients develop mild or no symptoms at all. For this reason, there is an emphasis on evaluating diagnostic tools for screening. Chest CT scans are emerging as a useful tool in the diagnostic process of viral pneumonia cases associated with COVID-19. This review examines the sensitivity, specificity, and feasibility of chest CT in detecting COVID-19 compared with real-time polymerase chain reaction (RT-PCR).

**Methods:**

Sensitivity and specificity of chest CT in detecting COVID-19 in its various phases was compared using RT-PCR as a gold standard. A “reverse calculation approach” was applied and treated chest CT as a hypothetical gold standard and compared RT-PCR to it point out the flaw of the standard approach.

**Results:**

High sensitivity (67–100%) and relatively low specificity (25–80%) was reported for the CT scans. However, the sensitivity of RT-PCR was reported to be modest (53–88%), hence cannot serve as an appropriate ground truth. The “reverse calculation approach” showed that CT could have a higher specificity (83–100%) if we consider the modest sensitivity of the RT-PCR.

**Conclusions:**

The sensitivity and specificity of the chest CT in diagnosing COVID-19 and the radiation exposure have to be judged together. Arguments are presented that chest CT scans have added value in diagnosing COVID-19 especially in patients, who exhibit typical clinical symptoms and have negative RT-PCR results in highly infected regions.

**Key Points:**

*• CT scans have higher specificity if we take into account the low sensitivity of the RT-PCR.*

*• Avoid chest CT as a sole diagnostic approach for COVID-19 infection.*

*• Patients who had negative RT-PCR result with typical clinical symptoms in highly infected regions or with close contact of COVID-19-infected patients; the use of chest CT is warranted.*

## Introduction

A novel coronavirus, named the severe acute respiratory syndrome coronavirus 2 (SARS-CoV-2) was identified in 2019 in China. The disease caused by the highly contagious virus is called the coronavirus disease 2019 (COVID-19). It spread over the world in a couple of months, causing high fatality and enormous burden on the health care providers. The identification of the infected patients has a high importance to control the disease. However the clinical picture might not be helpful, since a large majority of patients are asymptomatic or having only mild symptoms. Even if the patient had symptoms, those are rather unspecific (fever, cough, dyspnea). Consequently, the performance of various diagnostic tools such as molecular biological tests and imaging are in the focus of the current scientific interest. To date, one of the biggest questions is where chest CT stands in the screening and diagnostic process in comparison with real-time polymerase chain reaction (RT-PCR) test. In this paper, we try to summarize the results available so far in this topic. We evaluate the available data on the sensitivity, specificity, and accuracy of chest CT.

## Appearance of the disease on the chest CT

The SARS-CoV-2 is a member of the family of *Coronaviridae* and causes systemic and/or respiratory tract infections, rarely acute respiratory distress syndrome, or multi-organ failure. Its cellular entry is the angiotensin-converting enzyme 2 receptor which is expressed partly on the alveolar cells of the lung epithelium [1].

**Fig. 1:**
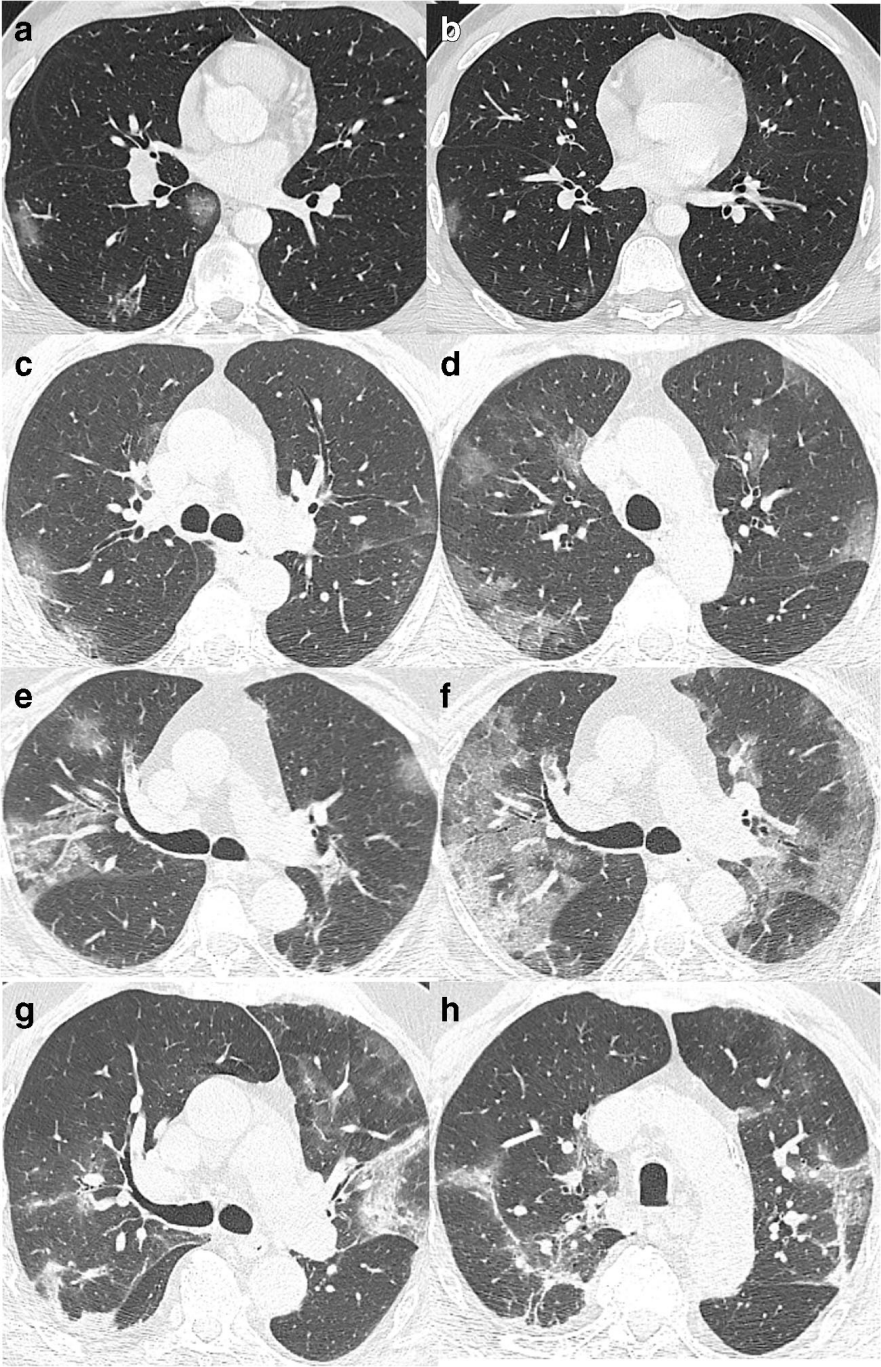
Appearance of COVID-19 on CT*.* **a***,* **b** A 35-year-old male presented 13 days after the symptom onset with unproductive cough, fatigue, and anosmia. Mild CT signs: GGOs in only one lobe. **c**, **d** A 60-year-old male having symptoms for 7 days: muscle pain, weakness, fever, and effort dyspnea. Bilateral, multilobar GGOs and halo sign (small consolidation surrounded by GGO) on the lower section. **e**, **f** A 73-year-old woman experiencing weakness, muscle pain, inappetence, and mild effort dyspnea. **e** Bilateral GGOs, thickened vessels, and traction bronchiectasis on the right. **f** 4 days later, the abnormalities are more extensive and crazy paving appeared within the GGO. **g**, **h** An 82-year-old man having symptoms for a week: dry cough, fever, weakness, inappetence, and low oxygen saturation at presentation. Several features are visible on the CT scans: GGOs, consolidation, organizing pneumonia with reverse halo sign

The CT manifestation of this viral pneumonia is non-specific but it has characteristic features based on which experienced radiologists can diagnose the disease. Findings are similar to other viral pneumonias but the localization of the signs is rather typical (see Fig. 1). The most frequent and earliest pattern is ground-glass opacity (GGO) that at first may be unifocal, but usually multifocal, bilateral, and peripheral distribution with a posterior predominance especially in inferior lobes. In the area of GGO, common findings are the widening of vessels and traction bronchiectasis [2].

The CT can be positive in the early phase, several days after the onset of the opening symptom (0–4 days). Over time, the CT findings change characteristically. In the progressive stage (5–8 days), the affected areas usually grow and sometimes thickened interlobular and intralobular lines appear inside the GGO. This combination pattern is called crazy paving. It is not characteristic for other viral pneumonias; hence, it can help the differential diagnosis [3].

The peak stage (9–13 days) is at about the 10th day. Consolidation often appears mixed with or after GGO. It can be seen as an early sign in elderly patients. The most severe clinical status is acute respiratory distress syndrome, which is radiologically equivalent to diffuse alveolar damage.

After that, in the absorption phase, organizing pneumonia pattern appears, and fibrous stripes can be seen with reverse halo sign and mild architectural distortion [3]. The abnormalities resolve in about 1 month.

## High sensitivity, but poor specificity of chest CT in the diagnosis of symptomatic patients

One of the largest case series on the correlation of chest CT and RT-PCR in COVID-19 is available from the epicenter of the outbreak in Wuhan [4]. The 1014 patients were assigned into three groups: The first two groups consisted of patients having typical clinical symptoms and positive chest CT with or without typical dynamic changes (81%). In the third group (19%), the patients had only one positive CT scan and presumably clinical symptoms (Table 1).

**Table 1:** Sensitivity, specificity, and accuracy of chest CT. RT-PCR gold standard

Publication	Country	Confirmed cases/1M*	Sensitivity	Specificity	Accuracy	Sample size	Days from symptom onset
[4] Ai T et al	China	19.49	97%	25%	68%	1014	N.R.
[5] Wu, J. et al	China	31.07	95%	N.A.		80	7 ± 4
[6] Himoto, Y. et al	Japan	6.9	67–83%	93–80%	86–81%	21	4-26
[7] Cheng, Z. et al	China	19.49	100%	25%	47%	38	1–9
[8] Caruso, D. et al	Italy	18.66	97%	56%	72%	158	N.R.

*N.R.* not reported, *N.A.* not applicable

*Prevalence data from https://ourworldindata.org/coronavirus at the time of data collection reported in the referenced papers

Eighty-eight of all patients had a positive initial chest CT. On the other hand, of all 1014 patients, only 601 (59%) had positive RT-PCR results. In these patients, the chest CT was positive in almost all cases (97%). In those patients who had negative RT-PCR result, chest CT was positive in 75%. If the authors considered the RT-PCR as a gold standard, the sensitivity, specificity, and accuracy of chest CT indicating COVID-19 infection were 97%, 25%, and 68%, respectively.

In another set of patients, also from Wuhan, 80 patients with clinical symptoms and positive RT-PCR were investigated. Chest CT was positive in 76 (sensitivity of 95%) patients [5].

In a study from Shanghai, China, on 38 suspicious COVID-19 patients (presumably all symptomatic), chest CT showed a sensitivity of 100%, specificity of 25%, and accuracy 47% [7].

A study from a non-high-epidemic area of Japan analyzed the results from 21 patients suspected to have COVID-19 at CT scans at least 3 days after symptom onset [6]. Six patients out of 21 had positive RT-PCR and 15 were proven to have other causes of the symptoms (Moraxella, legionella, pneumocystis, etc.). The sensitivity of the two raters was 67% and 83%, and the specificity 93% and 80%.

A study from Rome, Italy, also reported high sensitivity (97%) but moderate specificity (56%) of chest CT in comparison with RT-PCR in symptomatic patients [8]. Strengthening their results, RT-PCR was repeated within 24 h if negative on the first occasion.

Long presented data was from the city Yichang, China. In the study out of 87 symptomatic patients, who had both RT-PCR and chest CT performed, RT-PCR test was positive only in 36 cases. Out of the 36 patients, chest CT was normal only in one (sensitivity 97%) [9].

A recent meta-analysis, based on sixteen studies, calculated a pooled sensitivity of 92%. In their review, the authors identified only two studies [4, 10] reporting specificity (25–33%). Another meta-analysis found a pooled sensitivity of 94% and specificity of 37% [11].

## Sensitivity and specificity of RT-PCR

It was shown in a recent manuscript (submitted, not accepted at the time of the drafting of this manuscript) that the performance of the RT-PCR from various respiratory specimens is modest. During the first 2 weeks after symptom onset, 74–88% of sputum samples are positive and only 53–73% of the nasal swabs. Consequently, one might ask if RT-PCR can be considered as a gold standard. In the following section, we will re-evaluate the abovementioned studies as if the chest CT with typical clinical presentation was the reference (Table 2). This approach certainly underestimates the sensitivity of RT-PCR and overestimates the specificity of chest CT because of not considering other diseases having very similar clinical and CT presentation. However, the traditional estimation considering the RT-PCR as a gold standard (see previous chapter) might suffer from not considering the modest sensitivity of the RT-PCR.

**Table 2:** Sensitivity, specificity, and accuracy of RT-PCR according to the “reverse calculation” approach

Publication	Sensitivity	Specificity	Accuracy	Sample size	Days from symptom onset
[4] Ai, T. et al	65%	83%	67%	1014	N.R.
[7] Cheng, Z. et al	47%	100%	65%	38	1–9
[8] Caruso, D. et al	58%	96%	72%	158	N.R.

*N.R.* not reported

Starting with the largest study from Wuhan, if one considers the chest CT as a gold standard, the sensitivity of RT-PCR is 65%, the specificity is 83%, and the accuracy is 67%.

Considering Cheng’s study, if the chest CT was the reference, the sensitivity of PCR was only modest (47%) and obviously specificity is high (100%). Similarly, on the Italian sample, RT-PCR had a modest sensitivity and high specificity [8].

The study of Himoto offers an in depth insight into the performance of chest CT [6]. In this study, in the negative RT-PCR patients, alternative diagnosis was established. Unfortunately, not all patients had RT-PCR test for COVID-19 and only six of the 15 non-COVID patients had proven infectious agent other than SARS-CoV-2.

The collaboration of radiologists from Changsha (Hunan province of China) and Province, RI, USA, is providing a picture probably closer to the truth [12]. The authors collected chest CTs from 219 COVID-19 patients from China and 205 patients with positive Respiratory Pathogen Panel for viral pneumonia from Province, RI, from a time period between 2017 and 2019 when no COVID-19 was reported in the USA. The sensitivity for COVID-19 of the Chinese radiologists ranged from 72 to 94%, the specificity 24 to 94%, and the accuracy 60 to 83%. In a smaller subsample of patients, US radiologists had sensitivity of 70 to 93%, specificity of 93 to 100%, and accuracy of 84 to 97%.

Fang’s report from Eastern China showed that the sensitivity of the RT-PCR might not be optimal at the beginning of the disease. The first RT-PCR test performed in the first 3 (± 3) days after symptom onset was positive in only 36 of the 51 patients. Further 12 patients had positive RT-PCR test on the second occasion (24–48 h after the first), 2 patients by three tests (2–5 days), and one patient by four tests (7 days). On the other hand, 98% of the patients had positive chest CT scan on the first occasion (36 patients with typical and 14 with atypical CT manifestations) [13]. Similarly, in Long’s report, there were 36 patients having positive RT-PCR out of the 87 who had been included in the study [9]. However, 6 cases were missed on the first presentation with RT-PCR. Repeated RT-PCR test 48–72 h later identified further 3 patients and retest for the third time (5–8 days after the first) identified 3 patients. Importantly, the initial CT scan was positive in all but one patient. Therefore, the sensitivity of chest CT at the initial presentation was 97.2% and the RT-PCR only 84.6%. The importance of multiple RT-PCR tests were emphasized by Xie too [14].

It also has to be noted that the evolution of signs of the disease on chest CT is dynamic and peaks at around the 10th day after the first symptoms appeared [3]. Furthermore, it is also an important question when one should judge a chest CT positive. The experience of the reading radiologist may also influence the result diagnosing COVID-19. It is probably not a binary decision, but more of a spectrum to which a cutoff could be determined [15]; therefore, the already reported sensitivity and specificity values can be fairly over or underestimated. Similarly, the definition of positive chest CT can also influence the provided performance of RT-PCR.

## Chest CT in asymptomatic patients

The modest performance of RT-PCR testing raised the question whether it could be used in the early, asymptomatic stage of the disease and if chest CT could have an added value. Up to date, only a few reports presented data on this topic. Hu and co-workers showed that 50% of the asymptomatic SARS-CoV-2-infected patients had typical ground-glass opacities and further 20% atypical CT presentation [16]. Half of the CT-positive patients never developed any symptoms. Lin and colleagues reported about a patient having multiple GGOs in the right lung and not having clinical symptoms [17]. Later on, when the patient developed mild symptoms, the appearance of the CT scan changed accordingly. In the early publication by Shi and colleagues from Wuhan, 93% of the 15 preclinical patients had GGO on the chest CT [18].

In the homogenous cohort of Princess Diamond cruise ship, 73% of the 104 infected patients were asymptomatic. Fifty-four percent of these asymptomatic patients had lung opacities on chest CT. Asymptomatic cases showed more GGO than symptomatic patients, in whom the most frequent finding was consolidation [19].

## Feasibility of chest CT for screening COVID-19 infection

COVID-19 pandemic is spreading around the world with unprecedented speed. In this situation, it is important to analyze the available data to provide guidelines. From the studies we have reviewed above, it is clear that the chest CT has a high sensitivity to detect COVID-19. It seems, however, that while the identification of the virus RNA is the unanimous proof of the disease, the RT-PCR approach is not able to detect all the infections especially in the early phase of the disease. Even more, some studies suggest that chest CT might be more sensitive in this phase.

On the other hand, most of the studies so far suggested that the specificity of the chest CT is low. But it was compared with the performance of the RT-PCR, what is known to have a modest sensitivity. By selecting an inappropriate ground truth and consequently the number of true positives and negatives are being unknown, the calculation of performance will be erroneous. This fault in the literature was indicated by those studies which showed that multiple RT-PCR tests are increasing the detection rate of the disease. The higher specificity of chest CT was also showed by studies in which COVID-19 and other viral pneumonias were judged, by expert radiologists [12].

It also has to be seen that the specificity of chest CT is not as high as it is indicated by our above presented reverse calculation, because the ground truth is not known. But it is expected to be between the values presented earlier and the values from our reverse calculation.

To judge the feasibility of chest CT in COVID-19 screening, other factors have to be considered. In the use of chest CT as a first-line screening tool in large population, the risk-benefit ratio should be considered. Medical imaging is the largest man-made source of exposure that is about 0.6 mSv/year [20]. A standard-dose chest CT is in the range of 1.8 mSv, but low-dose protocol was shown to be effective identifying COVID-19 infection that has a dose about 0.2 mSv [21]. Introducing chest CT on a large-scale in the diagnosis of COVID-19 would increase the radiation exposure of the population significantly.

It also has to be considered whether the chest CT could increase the risk of transmission of the disease. With dedicated protection devices, the execution of the examination should not carry a higher risk for the medical staff than the risk during the swab test. With careful cleaning and appropriate air exchange rate, the nosocomial transmission of the disease can be avoided.

Importantly, CT suits with usual 6–8 air changes per hour require 35–45 min for airborne-contaminant removal with 99% efficiency. Similar time is needed for cleaning, making the throughput of a CT suit to 1–2 patients per hour.

Importantly, in regions where the prevalence of the disease is low, the introduction of chest CT into the screening protocol might not be warranted, since the relatively high specificity might only be true for regions with high occurrence of the disease. Moreover, this could certainly increase the unnecessary radiation exposure of the population.

After a careful consideration of the sensitivity, specificity, the risk of radiation exposure, and the throughput rate of chest CT and RT-PCR:

- We advise not to use chest CT as a sole diagnostic approach for COVID-19 infection.
- However, we think the use of chest CT in patients with typical clinical symptoms in highly infected regions or with close contact of COVID-19-infected patients who had negative RT-PCR result is warranted.
- The risk of radiation exposure probably outweighs the sensitivity of chest CT in asymptomatic patients.

## Abbreviations

COVID-19: Coronavirus disease 2019
GGO: Ground-glass opacity
RT-PCR: Real-time polymerase chain reaction
SARS-CoV-2: Severe acute respiratory syndrome coronavirus 2
